# Heat shock inhibits lipopolysaccharide-induced tissue factor activity in human whole blood

**DOI:** 10.1186/1477-9560-5-13

**Published:** 2007-09-24

**Authors:** Christoph Sucker, Kai Zacharowski, Matthias Thielmann, Matthias Hartmann

**Affiliations:** 1 Department of Haemostasis and Transfusion Medicine, Heinrich Heine University Medical Center, Dusseldorf, Germany; 2 Department of Anaesthesia, Bristol Royal Infirmary, Bristol, UK; 3 Department of Thoracic and Cardiovascular Surgery, University Hospital, Essen, Germany; 4 Department of Anaesthesiology and Intensive Care Medicine, University Hospital, Essen, Germany

## Abstract

**Background:**

During gram-negative sepsis, lipopolysaccharide (LPS) induces tissue factor expression on monocytes. The resulting disseminated intravascular coagulation leads to tissue ischemia and worsens the prognosis of septic patients. There are indications, that fever reduces the mortality of sepsis, the effect on tissue factor activity on monocytes is unknown. Therefore, we investigated whether heat shock modulates LPS-induced tissue factor activity in human blood.

**Methods:**

Whole blood samples and leukocyte suspensions, respectively, from healthy probands (n = 12) were incubated with LPS for 2 hours under heat shock conditions (43°C) or control conditions (37°C), respectively. Subsequent to further 3 hours of incubation at 37°C the clotting time, a measure of tissue factor expression, was determined. Cell integrity was verified by trypan blue exclusion test and FACS analysis.

**Results:**

Incubation of whole blood samples with LPS for 5 hours at normothermia resulted in a significant shortening of clotting time from 357 ± 108 sec to 82 ± 8 sec compared to samples incubated without LPS (n = 12; p < 0.05). This LPS effect was mediated by tissue factor, as inhibition with active site-inhibited factor VIIa (ASIS) abolished the effect of LPS on clotting time. Blockade of protein synthesis using cycloheximide demonstrated that LPS exerted its procoagulatory effect via an induction of tissue factor expression. Upon heat shock treatment, the LPS effect was blunted: clotting times were 312 ± 66 s in absence of LPS and 277 ± 65 s in presence of LPS (n = 8; p > 0.05). Similarly, heat shock treatment of leukocyte suspensions abolished the LPS-induced tissue factor activity. Clotting time was 73 ± 31 s, when cells were treated with LPS (100 ng/mL) under normothermic conditions, and 301 ± 118 s, when treated with LPS (100 ng/mL) and heat shock (n = 8, p < 0.05). Control experiments excluded cell damage as a potential cause of the observed heat shock effect.

**Conclusion:**

Heat shock treatment inhibits LPS-induced tissue factor activity in human whole blood samples and isolated leukocytes.

## Background

Gram-negative sepsis is mediated by lipopolysaccharide (LPS), a bacterial membrane constituent, which activates toll like receptor 4 (TLR-4). The resulting complex biological responses include an activation of the immune, inflammatory and coagulation systems [1-4]. While active tissue factor is absent in the peripheral blood under physiological conditions, the activation of hemostasis during sepsis is mediated by the expression of tissue factor on the surface of monocytes [5,6]. Intravascular tissue factor expression is of striking pathophysiological importance: the resulting activation of coagulation leads to disseminated intravascular coagulation, intravascular fibrin deposition, tissue ischemia and cell damage [7,8]. The importance of the coagulation system during sepsis is further highlighted by the fact, that recombinant activated protein C, a natural inhibitor of coagulation, is the only causative principle to improve the prognosis of this disease in humans [9].

There is strong evidence that temperature affects the immune response in humans and it has been suggested that fever might improve the prognosis of human sepsis [10-15]. In addition, it has been demonstrated that heat stress increases the survival rate subsequent to LPS-treatment in rats and reduces LPS-induced tumour necrosis factor (TNF) levels as well as vascular permeability in mice [16-18]. Therefore, we investigated whether heat shock affects LPS-induced activation of coagulation via a reduction of tissue factor expression.

## Methods

### Blood sampling

Venous blood was drawn from the antecubital vein of healthy volunteers (n = 12). After discarding the first 2 mL, blood was collected in one tenth volume of citrate (3.8 %, Becton Dickinson Vacutainer™) and samples were immediately used for the experiments. The ethical principles as set out in the Declaration of Helsinki were honored in the present study.

### Fractionation of whole blood samples

To obtain platelet poor plasma, whole blood aliquots were centrifuged at 2000 × g for 20 minutes. Absence of both leukocytes and platelets in platelet poor plasma was verified by transmission microscopy. Preparation of leukocytes was performed as recently described [19]. In short, 30 ml blood was drawn in a 50 ml syringe containing 5 ml of ACD-A (citrate 95 mmol/l, glucose 152 mmol/l, adenine). Thereafter, 6 ml hydroxyethylstarch (6 %) was added and red blood cells were allowed to sediment for 60 minutes. The cell rich supernatant was then centrifuged at 150 × g for 5 minutes. Thereafter, the leukocyte pellet was reconstituted in phosphate buffered saline (PBS) to 30.000 cells/μL and incubated as described below.

### Incubation of blood components with LPS

(i) For heat shock treatment, whole blood samples were incubated first at 43°C (2 hours) and then at 37°C (3 hours) with LPS (final concentration 100 μg/mL) or vehicle (NaCl 0.9 %). Furthermore, whole blood samples were incubated for 5 hours at 37°C with LPS or vehicle under otherwise identical conditions. Thereafter, clotting time of recalcified samples (400 μL) was determined using a KC 4 coagulometer (Amelung, Germany). Furthermore, both vehicle and LPS-treated whole blood samples were subjected to FACS analysis as described below. (ii) Identical incubation steps as outlined in (i) were performed with leukocyte suspensions instead of whole blood samples (final LPS-concentration was 100 ng/mL). In this series, clotting time was determined subsequent to the addition of three volumes citrated platelet poor plasma (to obtain a leukocyte count of 7500 cells/μL) and recalcification of the samples. Note, that the LPS concentration was thousand-fold lower in leukocyte suspension, as the reduced protein amount in these experiments reduces binding of LPS in comparison to whole blood experiments (for details: see discussion). Furthermore, the effects of heat shock and LPS on the cellular integrity of leukocytes was determined using the trypan blue exclusion test. Trypan blue 0.2 % was added to the suspensions for 20 minutes, thereafter cells were sedimented by centrifugation and reconstituted in PBS. The ratio of defect to intact leukocytes was calculated by judging trypan blue uptake of 500 leukocytes by light microscopy. (iii) To investigate the involvement of protein synthesis in the LPS-induced shortening of clotting time, whole blood samples were pretreated with cycloheximide (35 μg/mL) or vehicle for 30 minutes. Thereafter, LPS (100 μg/mL) or vehicle were added to the samples followed by incubation at 37°C for 5 hours and determination of clotting time. (iv) In a further series, active site-inhibited factor VIIa (ASIS, 50 μg/mL) was used to determine the importance of tissue factor for the observed LPS-effect on coagulation. Blood samples were incubated with LPS or vehicle for 5 hours at normothermia. Thereafter, ASIS was added and clotting time was measured ten minutes later [20].

### Flow cytometry

For immunolabeling of monocytes phycoerythrin-labelled mouse anti-CD 14 antibodies (BD Biosciences, Heidelberg, Germany) were used, leukocytes were identified using peridin chlorophyll labelled mouse antibodies against CD45 (BD Biosciences, Heidelberg, Germany). 5 μL of these antibodies were added to 10 μL citrate-anticoagulated blood in 35 μL PBS. Incubation time was 15 minutes. Immunolabeling was stopped by adding 1 mL PBS. For flow cytometry, a FACS Calibur (BD Biosciences, Heidelberg, Germany), gated for the detection of mononuclear cells, was used. WinMDI 2.8 (written by Joe Trotter) was used to present and calculate the results.

### Materials

LPS (Escherichia coli; serotype 0.111:B4) was obtained from Sigma-Aldrich, Germany. Hydroxyethylstarch (Voluven) was purchased from Fresenius Kabi, Germany. Active site-inhibited factor VIIa (ASIS) was a generous gift from Novo Nordisk, Denmark. All other reagents were of analytical grade.

### Statistics

All data are presented as mean and standard deviation. For statistical evaluation, the Mann-Whitney-Test was used and statistical significance was assumed with p-values below 0.05 (Openstat).

## Results

**Figure 1:**
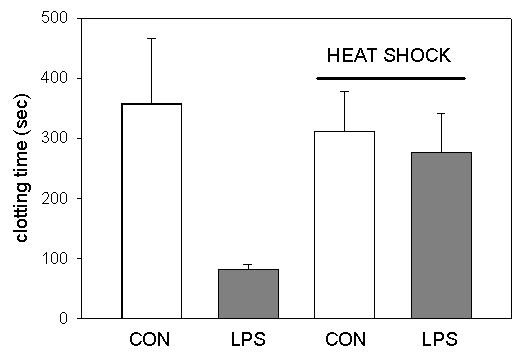
Effect of heat shock on the LPS-induced shortening of clotting time in whole blood samples. Whole blood samples, supplemented with LPS (100 μg/mL final concentration, LPS) or vehicle (CON), were incubated under heat shock conditions (2 hours at 43°C, 3 hours at 37°C) or normothermia (5 hours at 37°C). Thereafter, samples were recalcified and clotting time was determined. Results are shown as mean and standard deviation of 12 experiments per group. *: p < 0.05.

In a first series, we investigated the effects of LPS on clotting time: incubation of whole blood samples with LPS (100 μg/mL) shortened clotting time from 357 ± 108 sec to 82 ± 8 sec (n = 12; p < 0.05) when samples were incubated at 37°C for 5 hours (Figure 1). When samples were incubated for 2 hours at 43°C followed by 3 hours of incubation at 37°C, the LPS effect was completely abolished (Figure 1). Clotting time in whole blood samples treated with hyperthermia was 312 ± 66 sec in the absence of LPS (p < 0.05) and 277 ± 65 sec in the presence of LPS (p > 0.05).

**Figure 2:**
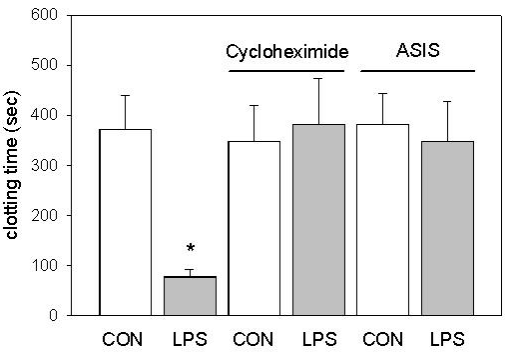
Effects of protein synthesis inhibition and tissue factor blockade on the LPS-induced shortening of clotting time. Whole blood samples were incubated with LPS (100 μg/mL) or vehicle (CON) in presence and absence of the protein synthesis inhibitor cycloheximide and the inhibitor of tissue factor effects, active site inhibited factor seven (ASIS), respectively. Results are shown as mean and standard deviation of 6 experiments per group. *: p < 0.05

The importance of protein synthesis for the LPS-induced shortening of clotting time was investigated in a further series. When whole blood samples were preincubated with the protein synthesis inhibitor cycloheximide (35 μg/mL), the effect of LPS on clotting time, determined 5 hours after incubation at 37°C, was blunted (Figure 2). To identify the LPS-induced clotting activity, active-site inhibited factor VIIa (50 μg/mL) was used. The LPS-induced activation of coagulation was completely inhibited in the presence of the tissue factor inhibitor (Figure 2). These results demonstrate that LPS exerts its action via de novo protein synthesis of tissue factor.

**Figure 3:**
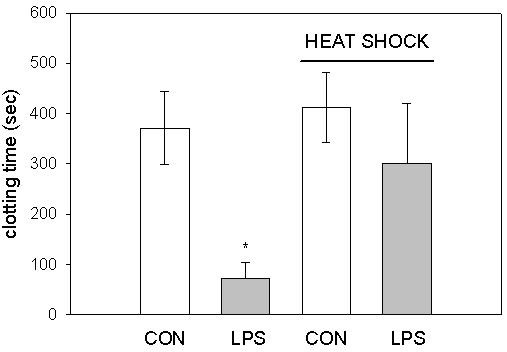
Effects of hyperthermia on LPS-induced tissue factor activity of leukocyte suspensions. LPS (100 ng/mL) or vehicle (CON) was added to freshly isolated leukocytes suspended in PBS, which were then incubated under heat shock conditions or normothermia. Thereafter, platelet poor plasma was added to the samples and clotting time as a measure of tissue factor was determined. Results are shown as mean and standard deviation of 8 experiments per group. *: p < 0.05

To determine the cell type which expresses tissue factor, we investigated the effects of hyperthermia on LPS-induced tissue factor expression in freshly isolated leukocytes. Leukocytes were incubated under normothermic or hyperthermic conditions in the presence or absence of LPS accordant to the protocol for whole blood experiments. Thereafter, platelet poor plasma was added and the clotting time as an indicator for tissue factor expression was measured (Figure 3). Under normothermic conditions, LPS shortened clotting time from 371 ± 72 s to 73 ± 31 s. Heat shock did not affect clotting time in absence of LPS (412 ± 70 s), but markedly reduced the clotting time in presence of the endotoxin (301 ± 118 s). The results demonstrate that expression of tissue factor in isolated leukocytes is inhibited by heat shock and that temperature dependent degradation of plasma constituents cannot explain the effects of hyperthermia. We used a lower LPS concentration in leukocyte experiments when compared to whole blood experiments (100 ng/mL vs. 100 μg/mL), because it is well known that LPS is incorporated into plasma proteins and erythrocyte membranes resulting in a far lower concentration of free endotoxin [21-25].

Various control experiments were performed to assure cell integrity after hyperthermia. Leukocyte count, which was in the range of 5400–8600/μL was not affected by hyperthermia and LPS. Furthermore, the rate of defect leukocytes, as determined by the trypan blue exclusion test, was not different under these conditions.

**Figure 4:**
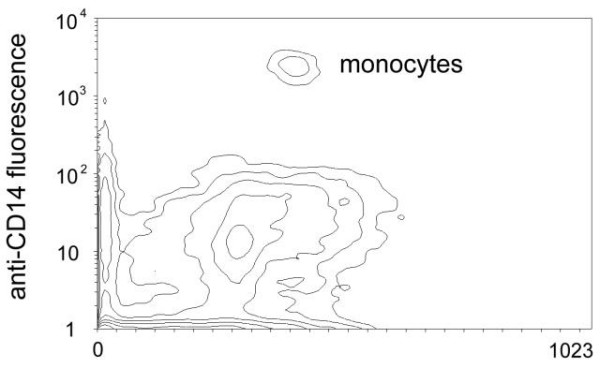
FACS analysis showing a contour plot of anti-CD14 fluorescence versus forward scatter. Monocytes are separated from other leukocytes by the more than 100-fold increase in fluorescence.

**Figure 5:**
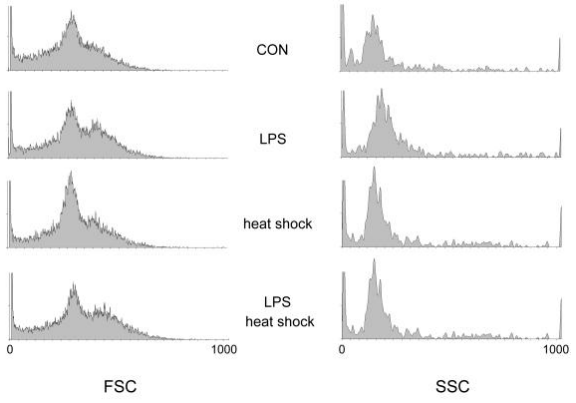
Distribution of forward and sideward scatter of monocytes treated with and without hyperthermia and LPS. Monocytes were identified by phycoerythrin-labelled anti-CD14 antibodies.

More specifically, the effects of hyperthermia and LPS-treatment on monocytes was investigated using FACS analysis, as this cell type represents the main source of tissue factor in the blood stream [5]. Monocytes were identified by labelling whole blood samples with fluorescent CD14-antibodies. A typical contour plot showing CD14-fluorescence and forward scatter of the FACS analysis is shown in Figure 4. Relation of monocytes to leukocytes was not affected by hyperthermia and LPS: relation of CD14 positive events to all events was in the range of 0.037 to 0.041. As a further proof of cell integrity, no difference in the forward- and sideward scatter characteristics of monocytes was observed, the amount of cell detritus, as judged from CD14-positive events with reduced forward scatter, was not different (Figure 5).

## Discussion

The present study indicates that hyperthermia inhibits the LPS-induced *de novo *synthesis of tissue factor in human whole blood and leukocyte suspensions. The effect of hyperthermia was demonstrated to be specific, because cellular integrity was not affected by heat shock treatment.

Tissue factor, an integral membrane protein, is the principle activator of coagulation *in vivo*. The protein is expressed on the surface of many cell types and initiates hemostasis in the case of vascular damage [26]. Under physiological conditions, active tissue factor is undetectable in the peripheral blood [6]. During sepsis, however, disseminated intravascular coagulation is a common finding and intravascular tissue factor expression is the major reason for septic coagulopathy. It has been demonstrated in recent studies that monocytes are the most important source of tissue factor expression during sepsis [5]. The disseminated intravascular coagulation is of striking pathophysiological importance as it leads to perfusion disturbances, tissue ischemia and septic organ dysfunction [7,8]. The importance of blood-borne tissue factor in the pathogenesis of sepsis is highlighted by the fact that administration of tissue factor pathway inhibitor and antithrombin exerts beneficial effects in animal sepsis models [3]. Furthermore, recombinant activated protein C proved to be the first pharmacological principle to reduce the mortality of sepsis in humans [9].

Since activation of coagulation induced by intravascular tissue factor contributes to the poor prognosis of sepsis, any intervention reducing the expression of tissue factor would be beneficial. There is good evidence that body temperature can influence the prognosis of sepsis [11-15].

Our study demonstrates that hyperthermia inhibits LPS-induced tissue factor formation in whole blood samples. Experiments were performed with whole blood samples, because important constituents of the complex network of coagulation and immune system are present in this model in physiological concentrations. Furthermore, monocyte count resembles *in vivo *conditions and an activation of cells by isolation steps cannot occur. We chose 2 hours of incubation for we aimed to mimick fever, which commonly lasts for a prolonged time. It is important to note that the effectivity of LPS in whole blood experiments is lower than in typical cell culture experiments. The reasons include binding of LPS to plasma proteins and red blood cells [21-23] Therefore, very low plasma concentrations (300 pg/mL, [24]) are accompanied by a huge LPS content of erythrocyte membranes (77 μg/mL, [25]). According to this fact, LPS induced tissue factor formation in our crude leukocyte suspension at a far lower concentration (100 ng/mL). The experiments with leukocyte suspensions demonstrated that heat shock inhibits LPS-induced tissue factor in cell suspensions devoid of plasma constituents, erythrocytes and thrombocytes. Thus, heat shock has a direct effect on leukocytes. An effect of hyperthermia and LPS on plasma components, e.g. degradation, can be excluded by this series because leukocytes were incubated in plasma free buffer and plasma was added after the incubation. For the determination of tissue factor we used a functional clotting assay in the present study. The advantage of this approach is that only functional active tissue factor is measured. In contrast, antibody based assays most likely detect non functional tissue factor fragments. Another often used activity based assay, which evaluates factor Xa generation in the presence of very high factor VIIa levels, detects soluble tissue factor, which has negligible activity at physiological factor VIIa levels (for details see [6]).

The degree of hyperthermia used in our study is commonly used for heat shock experiments and is also used for hyperthermic therapy in humans [17,18]. Most cell types (except nervous tissue) are not damaged by temperatures of 44°C [27]. For these reasons, cell damage cannot explain the observed inhibition of tissue factor activity. However, to further confirm the integrity of the leukocytes in our experiments, we performed several control experiments. Neither total leukocyte count nor trypan blue uptake were affected by heat shock. Moreover, flow cytometry did not reveal differences in monocyte morphology (as determined by forward and sideward scatter) and cell detritus formation.

Several studies demonstrate that other LPS-mediated effects are affected by hyperthermia. Heat shock treatment markedly reduced the LPS-induced increase in TNF-α in rats [17]. In mice, the LPS-induced increase in vascular permeability was inhibited by heat shock via a hsp90 dependent mechanism [18]. Furthermore, heat shock inhibited the LPS-induced IL-18 expression in murine macrophages [28]. Similar to our findings, heat shock reduced tissue factor activity and mRNA in human endothelial umbilical cells under in vitro conditions[29]. In addition, Egorina et al. 2006 demonstrated that rewarming of monocytes after hypothermia induces tissue factor expression, which, in turn, can be inhibited by heat shock treatment [30].

## Conclusion

In our study we were able to show that heat shock inhibits LPS-induced tissue factor activity in whole blood. We hypothesize that hyperthermia can reduce intravascular tissue factor formation during gram-negative sepsis. Studies to investigate the effect of fever on disseminated intravascular coagulation in patients with sepsis are warranted.

## Competing interests

The author(s) declare that they have no competing interests.

## Authors' contributions

CS, MT & MH : designed the study and performed the measurements. KZ: significantly added to the design of the study. All authors read and approved the final manuscript.
